# External auricle temperature enhances ear-based wearable accuracy during physiological strain monitoring in the heat

**DOI:** 10.1038/s41598-024-63241-2

**Published:** 2024-05-30

**Authors:** Shawn Chee Chong Tan, Trinh Canh Khanh Tran, Charis Yi Ning Chiang, Jieming Pan, Ivan Cherh Chiet Low

**Affiliations:** 1 Human Potential Translational Research Program, Yong Loo Lin School of Medicine, National University of Singapore, Singapore, Singapore; 2 Department of Physiology, Yong Loo Lin School of Medicine, National University of Singapore, Block MD9, 2 Medical Drive Level 4, Singapore, 117593 Singapore; 3 Department of Biomedical Engineering, National University of Singapore, Singapore, Singapore; 4 Department of Electrical and Computer Engineering, National University of Singapore, Singapore, Singapore

**Keywords:** Machine learning, Translational research, Occupational health, Public health

## Abstract

Body core temperature (T_c_) monitoring is crucial for minimizing heat injury risk. However, validated strategies are invasive and expensive. Although promising, aural canal temperature (T_ac_) is susceptible to environmental influences. This study investigated whether incorporation of external auricle temperature (T_ea_) into an ear-based T_c_ algorithm enhances its accuracy during multiple heat stress conditions. Twenty males (mean ± SD; age = 25 ± 3 years, BMI = 21.7 ± 1.8, body fat = 12 ± 3%, maximal aerobic capacity (VO_2max_) = 64 ± 7 ml/kg/min) donned an ear-based wearable and performed a passive heating (PAH), running (RUN) and brisk walking trial (WALK). PAH comprised of immersion in hot water (42.0 ± 0.3 °C). RUN (70 ± 3%VO_2max_) and WALK (50 ± 10%VO_2max_) were conducted in an environmental chamber (T_db_ = 30.0 ± 0.2 °C, RH = 71 ± 2%). Several T_c_ models, developed using T_ac_, T_ea_ and heart rate, were validated against gastrointestinal temperature. Inclusion of T_ea_ as a model input improved the accuracy of the ear-based T_c_ algorithm. Our best performing model (T_rf3_) displayed good group prediction errors (mean bias error = − 0.02 ± 0.26 °C) but exhibited individual prediction errors (percentage target attainment ± 0.40 °C = 88%) that marginally exceeded our validity criterion. Therefore, T_rf3_ demonstrates potential utility for group-based T_c_ monitoring, with additional refinement needed to extend its applicability to personalized heat strain monitoring.

## Introduction

Climate change has precipitated elevations in global surface temperatures and increased incidences of extreme weather events, which causes detrimental effects to human health and performance. Greater frequency of intense heatwaves between 2017 to 2021 have culminated in a 68% upsurge in heat-related mortalities within susceptible populations, comprising of older adults and young children below the age of one^1^. This is also a concern for individuals in numerous heat-exposed occupations (e.g. military personnel, emergency responders and manual workers) who typically perform tasks involving substantial physical workloads whilst donning thick personal protective equipment (PPE). Furthermore, major international sporting events, such as the Summer Olympics and the Track-and-Field World Championships, often take place during the hottest periods of the year^2^. With climate change, athletes face increasingly hot ambient conditions during training and competitions which impairs exercise performance and elevates their risk of developing exertional heat stroke^3,4^. Despite extensive documentation on the prevention and treatment of exertional heat stroke, its prevalence continues to grow^5–8^. This suggests that current heat strain management strategies remain inadequate to fully tackle the problem at hand.

Existing heat strain management strategies centre on the identification of high-risk environments and behavioural modifications based on environmental heat stress^9^. However, these strategies fail to consider crucial predisposing factors such as individual differences in metabolic heat production, physical fitness, heat acclimatisation/acclimation (HA) status and heat injury history^10^. The implementation of personalized physiological monitoring, using wearable technology, is a potential solution to account for individual thermal strain^11,12^. This is achieved through continuous monitoring of various physiological parameters; including body core temperature (T_c_), heart rate (HR), skin temperature (T_sk_) and sweat loss^5^. Personalized physiological monitoring can complement existing strategies by improving work rest cycle development and individualized safety monitoring.

Particularly, accurate prediction of T_c_, either prospectively or in real-time, may be crucial in preventing over- or under-protection from heat-related illness^9,13^. Yet, there are currently no accurate and practical methods for monitoring T_c_ in occupational and/or athletic settings. Although rectal or oesophageal thermistors are valid for continuous monitoring of human T_c_, such sensors are highly invasive, single-use, and can cause significant user discomfort thus making them unfeasible for daily implementation^14^. Furthermore, despite an improved user comfort when utilising ingestible telemetric pills, this strategy comes with a prohibitively high cost and is complex to implement due to the need to account for individual differences in gastrointestinal motility^9^. While non-invasive surrogates such as measurement of oral and axilla temperature have been implemented for recording of T_c_ in clinical settings, these strategies remain unsuitable for use during physical activity due to a high susceptibility to environmental factors and inability to provide continuous T_c_ measurement^14^.

Amongst the host of measurement sites explored, the ear has notably emerged as a viable option for human T_c_ measurement. Tympanic membrane temperature (T_ty_) was proposed due to the vascularisation of the tympanic membrane by the internal carotid artery which also irrigates the hypothalamus^15^. Measurement of T_ty_ is achieved by direct contact with the tympanic membrane or indirect measurement of heat emitted from the tympanic membrane and aural canal^16^. While the former demonstrated acceptable correlations with T_c_^17^, it is unsafe for use in exertional heat strain monitoring as shifting of the thermistor during physical movement can lead to tympanic membrane injury or cause pain should the sensor contact the richly innervated portion of the aural canal^16^. Indirect T_ty_ measurement using infrared sensors provides better comfort and safety. However, as a line of sight to the tympanic membrane is necessary for accurate reflection of T_c_, factors such as aural canal shape and/or inadequate depth of insertion can lead to discrepancies^18^.

Monitoring of aural canal temperature (T_ac_) is a promising alternative. Indeed, T_ac_ measurements displayed good correlation with rectal temperature (T_re_) when the sensor was placed 10 mm away from the tympanic membrane^19^. Furthermore, Nagano, et al.^20^ demonstrated small deviations between T_ac_ and T_re_ during intermittent cycling. This is further supported by recent findings which reported that oesophageal temperature (T_es_) was reliably predicted following modelling of T_ac_ inputs from multiple sensors along the aural canal^21^. Importantly, no subject discomfort was reported as a result of the sensor placement^16,20,22^ which supports the notion that T_ac_ monitoring can be an ideal method for monitoring of heat strain.

Despite its promise, the development of an algorithm based on T_ac_ inputs alone does have its limitations. Prediction of T_c_ based on T_ac_ inputs alone is challenging as the accuracy of T_ac_ measurements can be influenced by variations in ambient temperature^20^. In this regard, we postulate that changes in external auricle temperature (T_ea_) and HR can be incorporated into the wearable ear-based algorithm to account for the effect of ambient temperature and metabolic heat on T_ac_. The external auricle (site of measurement for T_ea_) consists largely of skin and cartilage^23^ and is thus unable to generate metabolic heat due to a lack of skeletal muscle tissue. As such, changes in T_ea_ would primarily stem from dissipation and/or absorption of heat from the surrounding environment. This allows the external auricle to serve as a suitable measurement site to account for the effect of different ambient conditions on T_ac_. Thus, the inclusion of T_ea_ as additional physiological variable harbours the potential to enhance the predictive accuracy of an ear-based T_c_ algorithm.

To achieve this, we modified a commercially available T_ac_-measuring ear-based wearable with an additional sensor for T_ea_ measurement. We then sought to develop an algorithm to predict T_c_ during passive- and exercise-induced heat stress by using T_ea_ and a host of other physiological variables from the ear-based wearable as inputs for model development. Finally, we evaluated the validity of the algorithm developed for non-invasive heat strain monitoring under hot and humid environmental conditions.

## Methodology

### Participants

Twenty healthy physically active males (mean ± SD; age = 25 ± 3 years, BMI = 21.7 ± 1.8, body fat = 12 ± 3%, maximal aerobic capacity (VO_2max_) = 64 ± 7 ml/kg/min) were recruited for this study. Participants were native to Singapore and had a 10-km run time of less than 60 min. Only individuals certified fit for participation by an independent medical practitioner, with no existing musculoskeletal injury, anal piles or respiratory diseases, and/or history of digestive tract surgery, heat injuries or heart diseases were recruited.

All procedures were approved by the Institutional Review Board of the National University of Singapore (reference number: H-20-017) in accordance with the Declaration of Helsinki. The purpose, procedures, benefits and risks of the study were verbally explained, and participants provided their written informed consent prior to participation.

## Experimental design

**Figure 1:**
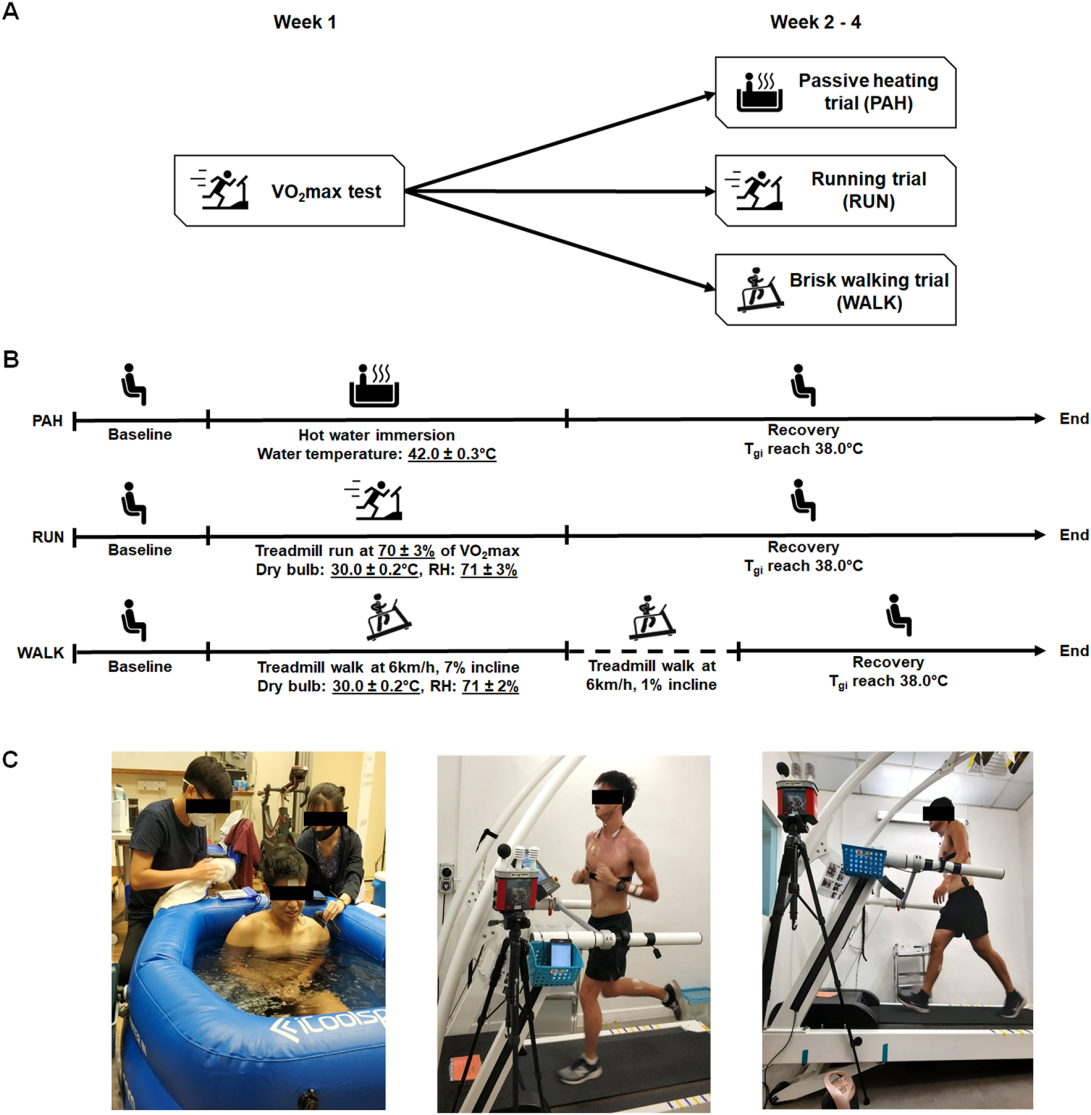
(**A**) Schematic representation of the overall study design, (**B**) experimental trial design and (**C**) experimental trial photos (from left: PAH, RUN, WALK). Participants performed a seated baseline. During PAH, participants immersed themselves up to chest level in water maintained at 42.0 ± 0.3 °C. During RUN, participants ran on a motorised treadmill at a speed that corresponded to 70 ± 3% of their VO_2max_. During WALK, participants performed a treadmill walk at a speed of 6 km/h with an elevation of 7%. Passive and/or exercise-induced heating was terminated when participants’ T_gi_ reached 39.5°C. During WALK, participants that did not achieve the target T_gi_ within a 60 min duration underwent an extended exercise phase. This consisted of a treadmill walk at a speed of 6 km/h with an elevation of 1%, for a maximum duration of 30 min. Subsequently, participants underwent a seated recovery until T_gi_ returned below 38.0 °C. *VO*_*2*_*max* Maximal aerobic capacity, *RH* Relative humidity, *T*_*gi*_ gastrointestinal temperature.

Participants performed a VO_2max_ test on the first laboratory visit to assess their aerobic fitness and to individualize the exercise intensity employed in subsequent trials. Anthropometric measurements were also recorded on their first visit. Subsequently, participants underwent three experimental trials: a passive heating (PAH), a running (RUN) and a brisk walking (WALK) trial (Fig. 1C). Three different modes of heating/exercise were employed to facilitate the development of a robust ear-based T_c_ algorithm, with broad applicability over a variety of activities and exercise intensities. Participants completed all experimental trials in a randomly assigned order (Fig. 1A).

### Anthropometric measures

Nude body mass and height were recorded using a floor weighing scale (BBA211 Bench Scale, Mettler-Toledo, Germany) and a stadiometer (Seca, Brooklyn, NY, USA) respectively. Body mass index (BMI) was calculated as (body mass in kg)/(height in m)^2^. Skin folds were measured from four sites (bicep, tricep, subscapular and suprailiac) using a Harpenden skinfold calliper (Model HSK-BI; British Indicators, West Sussex, UK). Skin fold measurements from these four sites were necessary to estimate body surface area^24^, body density^25^ and body fat percentage^26^.

### Maximal aerobic capacity (VO_2max_) test

An incremental treadmill protocol was used to measure each participant’s VO_2max_^27^. The first phase consisted of a treadmill run at four different speeds, with an initial speed that was 1 km/h slower than the participant’s expected 10 km race pace. Treadmill speed was increased by 1 km/h every three min, for a total duration of 12 min. Following a five min rest, participants proceeded to the second phase which consisted of a treadmill run at a fixed individualized speed of moderate intensity (treadmill speed ranged from 9 km/h to 12 km/h as determined by the researcher based on the previous phase), with an initial elevation of 1%. Treadmill elevation was increased by 1% every min until participants reached volitional exhaustion. Oxygen uptake (VO_2_) was measured using a metabolic cart (TrueOne 2400, Parvo Medics East Sandy, UT, USA; accuracy ± 0.1%) and VO_2max_ was derived from the mean VO_2_ measured during the final minute prior to test termination.

### Experimental trials

Participants were requested to avoid alcoholic beverages, have at least eight hours of sleep, consume sufficient water to stay hydrated and repeat a similar diet and any physical activity performed 24 h prior to each experimental trial. To facilitate their compliance with the study requirements, participants completed a 24-h dietary and physical activity questionnaire. Participants provided a mid-stream urine sample for measurement of urine specific gravity (USG) using a refractometer (UG-alpha, Atago, Bellevue, WA, USA). All participants were euhydrated (USG = 1.000 to 1.024) prior to the commencement of the trials (USG < 1.025^28^).

**Figure 2:**
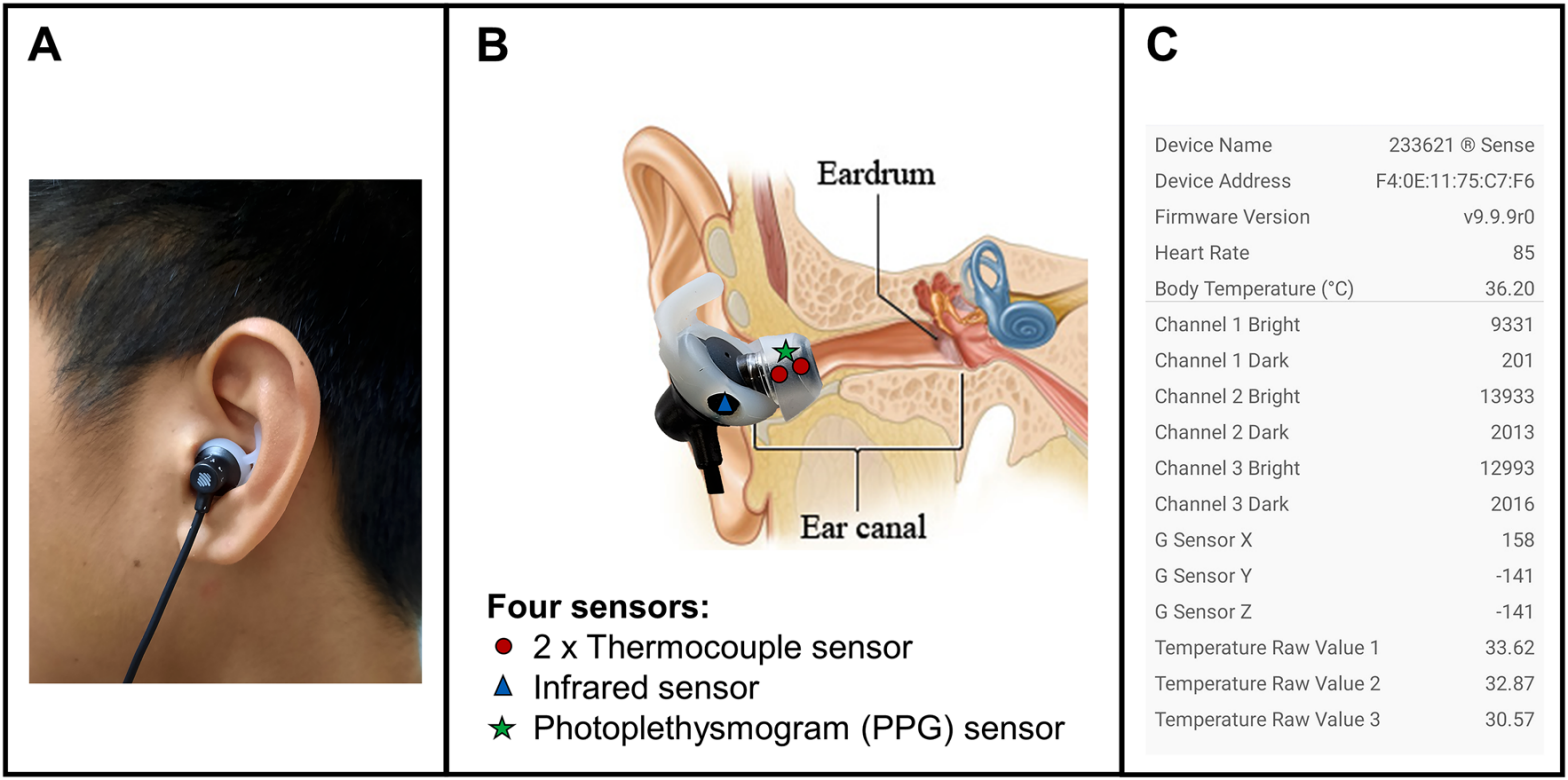
(**A**) The ear-based wearable device placed in a participant’s ear. (**B**) Schematic representation of sensor placement on the ear. Aural canal temperature was measured by two thermocouple sensors while external auricle temperature and heart rate were measured by an infrared sensor and a photoplethysmography (PPG) sensor respectively. (**C**) Logging of physiological parameters on mobile application.

Gastrointestinal temperature (T_gi_) was monitored using an ingestible telemetric sensor (e-Celsius®, BodyCap, Hérouville-Saint-Clair, France) with a sampling rate of 15 s. Owing to its established validity when compared against rectal and oesophageal temperature^29^, T_gi_ was utilized as the gold standard reference for T_c_ in the present study. The telemetric sensor was either ingested eight to ten hours before each session or rectally inserted by participants upon arrival at the trial site. Heart rate (HR) was continuously measured every second by a chest-based monitor (M430 with H10 HR monitor, Polar Electro, Kempele, Finland). An ear-based wearable device (233621 Sense Headphones, Grandsun Electronic Co. Ltd, Shenzhen, China) was utilized to collect data for model development (Fig. 2A). The device continuously measured aural canal temperature (T_ac_) using two thermocouple sensors (T_ac1_ and T_ac2_, maximal error of ± 0.1 °C between − 20 °C to 50 °C^30^) and HR using a photoplethysmography (PPG) sensor (HR_ear_) (Fig. 2B). In addition, a commercial earpiece was modified by adding an infrared (IR) sensor which was placed in close proximity to the skin to measure external auricle temperature (T_ea_, Fig. 2B). IR thermometry is commonly preferred over thermocouples for the measurement of peripheral skin temperature in clinical settings as IR sensors are not required to be in continuous contact with the skin^31^. This feature is especially important in instances where thermometry is performed during constant movement or physical activity. T_ac1_, T_ac2_, T_ea_ and HR_ear_ data were transmitted to a mobile application via Bluetooth and logged every second (Fig. 2C). VO_2_ was measured at baseline and at 15-min intervals during RUN and WALK. Additionally, every 15 min, participants were provided with 2 g/kg body mass of ambient water maintained at 26.0 °C to prevent hypohydration (> 2% reduction in body mass due to water loss^32^) during the trials. The experimental trial design is depicted in Fig. 1B.

### Passive heating trial (PAH)

Participants donned running shorts and completed a 10 min seated baseline in an airconditioned laboratory environment (dry bulb temperature (T_db_) = 21.6 ± 0.5 °C, relative humidity (RH) = 68 ± 3%, wet-bulb globe temperature (WBGT) = 19.2 ± 0.5 °C). Subsequently, participants immersed themselves up to chest level in an inflatable tub containing water maintained at 42.0 ± 0.3 °C by an external heating unit (Compact XP Dual Temp, iCoolsport, Gold Coast, Australia). Light facial fanning was applied during heating to minimize participant discomfort. Participants were passively heated until either T_gi_ of 39.5 °C or total duration of 60 min was reached. Following completion of the heating phase, participants underwent a seated recovery until T_gi_ returned below 38.0 °C.

### Running trial (RUN) and brisk walking trial (WALK)

The RUN and WALK trials were conducted in a controlled environmental chamber set to simulate a warm and humid tropical environment (T_db_ = 30.0 ± 0.2 °C, RH = 71 ± 2%, WBGT = 27.1 ± 0.3 °C). Participants donned running attire with sports shoes and completed a 10 min seated baseline prior to commencement of the exercise. In RUN, participants exercised on a motorized treadmill (h/p/cosmos Mercury, Germany) at a speed that corresponded to 70 ± 3% of their VO_2max_. In WALK, participants performed a treadmill walk at a speed of 6 km/h with an elevation of 7%. The exercise was terminated if participants’ T_gi_ reached 39.5 °C. Participants whose T_gi_ were still below that safety threshold after 60 min underwent an extended exercise phase to elicit a further rise in T_gi_. The extension was a treadmill walk at a speed of 6 km/h with an elevation of 1%, for a maximum duration of 30 min. Subsequently, participants underwent a seated recovery until T_gi_ returned below 38.0 °C.

### Model development

Physiological data recorded by the ear-based wearable (T_ac1_, T_ac2_, T_ea_ and HR) were used as base parameters for data modelling. All base parameters were pre-processed into 15 s averages and time aligned with T_gi_ data from the telemetric capsule. The temperature gradient (T_grad_) between the internal and external regions of the ear was computed as a parameter that accounts for heat exchange between the environment and the aural canal. T_grad_ was quantified by the following equation:

$${T}_{grad}=\frac{{T}_{ac1} + {T}_{ac2}}{2}- {T}_{ea}$$

Feature engineering was undertaken to generate new modelling parameters from the base physiological parameters and modality parameters. While physiological parameters are continuous, modality parameters are categorical data indicating the activity modalities (passive heating, running, walking) and the phase of trial (pre-trial baseline, heating, post-trial recovery). The feature engineering methods employed encompassed mathematical transformations, linear regression transformations, polynomial regression transformations up to order three, data segmentation. Data smoothening techniques, namely Savitzky-Golay filter and rolling average, were employed to reduce noisy data and improve overall signal-to-noise ratio.

Three regression algorithms, namely linear regression (T_lin_), second-order polynomial regression (T_poly_) and random forest regressor (T_rf_) were evaluated in the study. These algorithms were selected for their reported potential to predict T_c_ from various physiological parameters^20,21,33^. For each algorithm, an iterative feature selection approach was employed to compare the model performances with different subsets of parameters. Algorithm development was performed with machine learning package Scikit-learn on Python version 3.10.

Five-fold cross-validation technique was employed, where training was repeated five times with different training subsets. At each fold, the training dataset consisted of 75% of the subjects, and the testing dataset consisted of the remaining subjects. The performance of each model was averaged from all five folds to minimise any random biases. To assess the performance of the models, the selected evaluation metrics are mean bias error (MBE), mean absolute error (MAE) and 95% confidence intervals (CI). This set of metrics captures accuracy, precision, and reliability of individual estimates respectively. Optimal model performance is characterised by smaller values of these metrics, signifying the predicted values are good estimates of T_c_.

### Data analysis

All statistical computations were performed using IBM SPSS Statistics version 29 (IBM SPSS Statistics 29.0, Armonk, NY, USA) and figures were produced using GraphPad Prism version 10.0.0 (GraphPad Software, San Diego, CA, USA). Normality of data were evaluated using a Shapiro–Wilk test. Bland–Altman plots were used to assess for the agreement between ear-based wearable data and gold standard references. The MBE was calculated by subtracting ear-based wearable data from gold standard references at each 15 s time-point and subsequently averaging all errors. The MAE was quantified by averaging all absolute errors. The 95% CI were calculated as 1.96 × standard deviation (SD) of errors. Percentage target attainment of errors within ± 0.4 °C (PTA ± 0.4 °C) were quantified for the ear-based T_c_ algorithm(s). RMSE was calculated as the square root of the mean of the total squared bias between estimated T_c_ and T_gi_. The degree of correlation was determined as follows: very strong (r > 0.90), strong (r = 0.70 to < 0.90), moderate (r = 0.50 to < 0.70), low (r = 0.30 to < 0.50) and negligible (r < 0.30)^34^. The following criterion were used to determine the validity of the ear-based T_c_ algorithm for prediction of T_gi_: (a) individual prediction errors: 95% PTA within ± 0.40 °C^29^, (b) group prediction errors: MBE <  ± 0.27 °C^35^. Mean absolute percentage error (MAPE) and two-way mixed-effects Intraclass Correlation Coefficient (ICC) were calculated to assess the accuracy of the ear-based HR sensor. ICC was determined accordingly: excellent (> 0.90), good (> 0.75 to 0.90), moderate (0.50 to 0.75), poor (< 0.50)^36^. Validity of the ear-based HR sensor was determined by a MAPE < 10%^37^ and ICC > 0.90. All data were presented in mean ± SD.

## Results

Data were collected across 60 experimental trials (20 participants completed three trials each). However, ear-based wearable data were unavailable during eight trials due to battery and/or intermittent connectivity issues. These incomplete datasets were excluded from data modelling and analysis. Thus, the ear-based T_c_ algorithm was developed and evaluated across 52 trials.

A wide range of T_gi_ and HR measurements were recorded during the three experimental trials as intended by our study design. The T_gi_ dataset consisted of 18,592 data points (15 s averages) ranging from 36.4 to 40.0 °C while the HR dataset comprised of 32,816 data points (5 s averages) ranging from 45 to 201 bpm. The ear-based PPG HR sensor met both validity criterion implemented in the present study as demonstrated by an acceptable MAPE of 2.1 ± 3.4% and an excellent ICC of 0.992. Participants reached the study’s T_gi_ cutoff in 22 trials (PAH = 9, RUN = 12, WALK = 1).

### Agreement between T_ac_ and T_gi_

**Figure 3:**
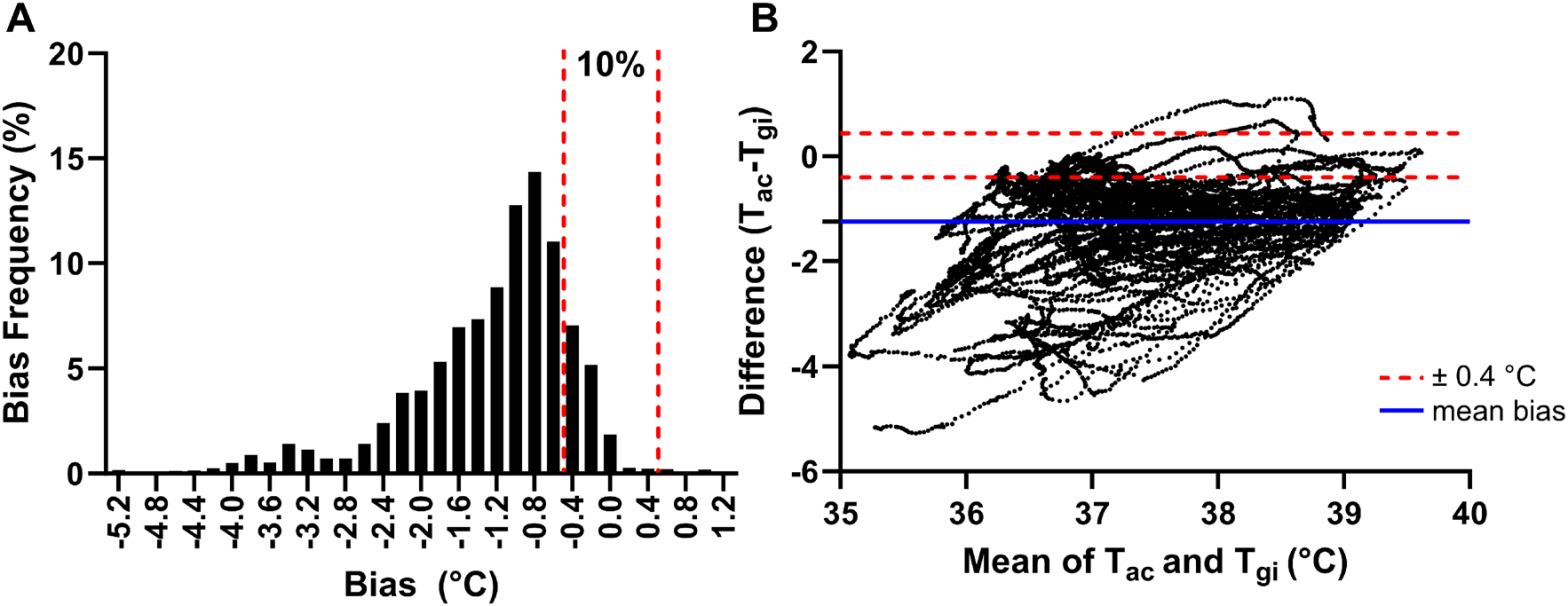
(**A**) Histogram depicting percentage distribution of errors and (**B**) Bland–Altman plots comparing aural canal temperature (T_ac_) data against the telemetric capsule (T_gi_) across all trials. The solid blue line represents the mean bias error while the red dashed lines represent fixed upper and lower limits of agreement of ± 0.40 °C.

The agreement between T_ac_ and T_gi_ was assessed to determine the validity of T_ac_ as a surrogate measure of T_c_. Both PTA ± 0.40 °C (10%) and MBE (-1.25 ± 0.86 °C) did not meet the validity criterion set in the present study (Fig. 3). Moreover, 95% CI (± 1.69 °C) was large when comparing between T_ac_ and T_gi_ (Fig. 3).

### Model selection and parameter importance

To identify prediction models capable of enhancing the accuracy of T_c_ predictions derived from T_ac1_, T_ac2_, T_ea_ and HR inputs, we compared a linear regression model (T_lin_), second order polynomial regression model (T_poly_) and random forest regressor model (T_rf1_). The ear-based HR data used for data modelling in our study met both validity criteria, as indicated by an acceptable MAPE of 2.1 ± 3.4% and an excellent ICC of 0.992.

**Figure 4:**
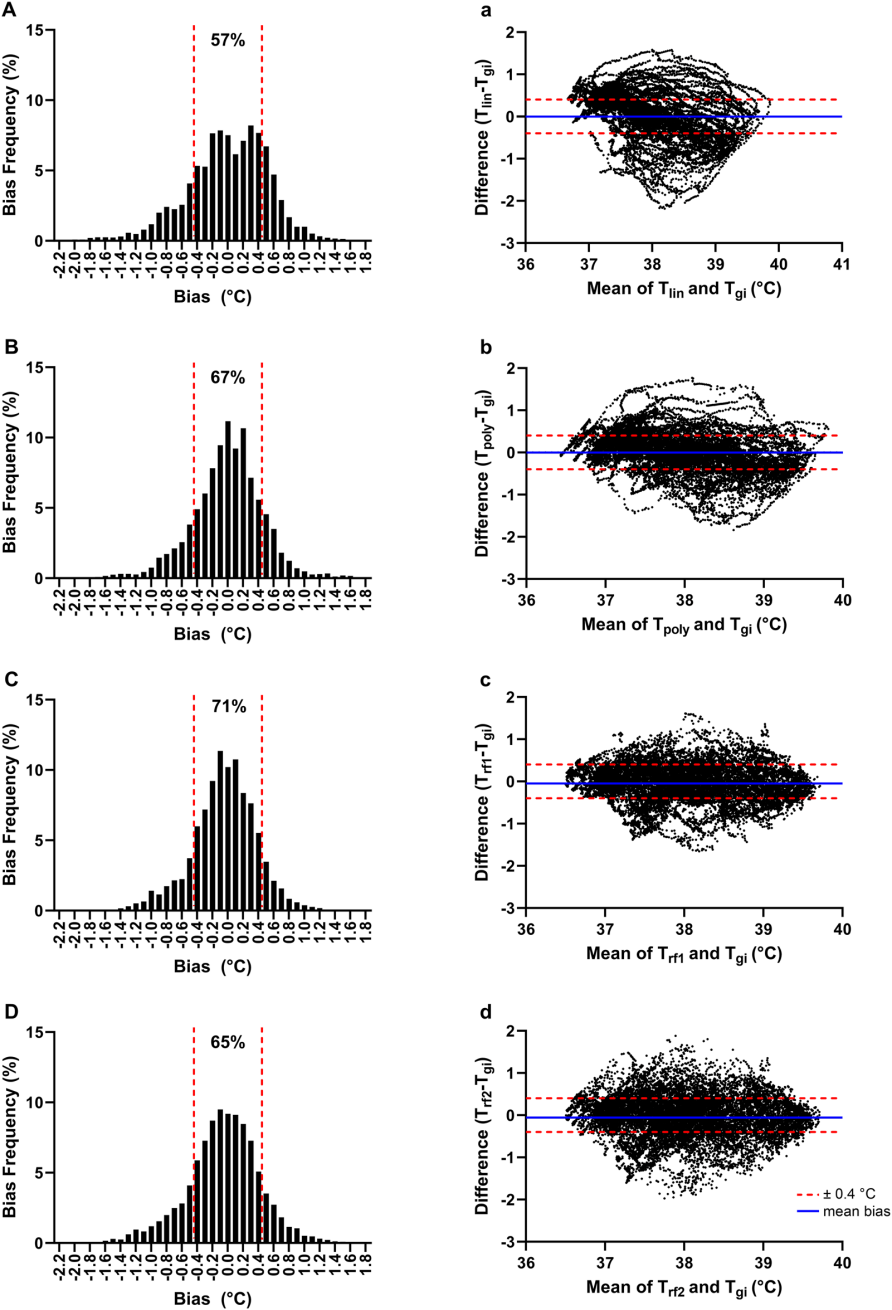
Histogram depicting percentage distribution of errors when comparing T_c_ data predicted by (**A**) linear regression model (T_lin_), (**B**) polynomial regression model (T_poly_), (**C**) random forest regressor model including T_ea_ data (T_rf1_) and (**D**) random forest regressor model excluding T_ea_ data (T_rf2_) against the telemetric capsule (T_gi_) across all trials. Bland–Altman plots comparing T_c_ data predicted by (**a**) linear regression model (T_lin_), (**b**) polynomial regression model (T_poly_), (**c**) random forest regressor model including T_ea_ data (T_rf1_) and (**d**) random forest regressor model excluding T_ea_ data (T_rf2_) against the telemetric capsule (T_gi_) across all trials. The solid blue line represents the mean bias error of each model while the red dashed lines represent fixed upper and lower limits of agreement of ± 0.40 °C.

The MBE for T_lin_ (0.00 ± 0.53 °C), T_poly_ (0.00 ± 0.46 °C) and T_rf1_ (-0.05 ± 0.42 °C) were within acceptable limits (Fig. 4a–c), but PTA ± 0.40 °C did not meet our pre-determined validity criterion (all < 95%, Fig. 4A–C). T_rf1_ displayed the highest PTA ± 0.40 °C (71%) and narrowest 95% CI (T_lin_ =  ± 1.04 °C, T_poly_ =  ± 0.89 °C, T_rf1_ =  ± 0.82 °C, Fig. 4a–c) among the three prediction models. Additionally, omitting T_ea_ data as an input in the random forest regressor model (T_rf2_) resulted in larger individual prediction errors, as evidenced by a reduced PTA ± 0.40 °C (65%, Fig. 4D) and a larger 95% CI (± 0.94 °C, Fig. 4d) compared to T_rf1_.

### Parameter engineering

Physiological parameters measured by the ear-based wearable only displayed moderate correlations (r = 0.34–0.56) with T_gi_, which could explain the sub-optimal performances observed from the selected T_c_ prediction models (Fig. 4). Interestingly, we found that T_ac1_ + T_grad_, which accounts for the gradient between internal and external temperature at the ear, displayed strong correlation with T_gi_ (r = 0.77). T_grad_ calculated in the present study ranged from 0.0 to 4.7 °C. Hence, feature engineering was performed using these basic parameters to generate additional highly correlated model inputs for data modelling. Sixteen new parameters were developed, each demonstrating strong to very strong correlations with T_gi_ (Table 1).

**Table 1:** Correlation, mean absolute error and 95% CI between basic parameters (T_ac1_, T_ac2_, T_ea_, HR, T_ac1_ + T_grad_) and engineered data modelling parameters, against telemetric capsule (T_gi_) across all trials.

	Correlation (r)	MAE (°C)	95% CI (°C)
Basic parameters:			
T_ac1_	0.56	1.26	± 1.69
T_ac2_	0.49	1.54	± 1.93
T_ea_	0.34	2.60	± 3.22
HR	0.42	Not applicable	Not applicable
T_ac1_ + T_grad_	0.77	0.42	± 1.01
Engineered parameters:			
T_eng1_	0.82	0.26	± 0.65
T_eng2_	0.82	0.22	± 0.56
T_eng3_	0.86	0.30	± 0.74
T_eng4_	0.88	0.27	± 0.69
T_eng5_	0.88	0.26	± 0.65
T_eng6_	0.90	0.36	± 0.90
T_eng7_	0.90	0.24	± 0.61
T_eng8_	0.91	0.32	± 0.78
T_eng9_	0.91	0.25	± 0.63
T_eng10_	0.92	0.42	± 1.04
T_eng11_	0.92	0.35	± 0.88
T_eng12_	0.92	0.25	± 0.63
T_eng13_	0.92	0.20	± 0.52
T_eng14_	0.93	0.30	± 0.75
T_eng15_	0.94	0.34	± 0.89
T_eng16_	0.95	0.20	± 0.52

### Validity of ear-based T_c_ algorithm

To derive the best performing T_c_ prediction model, we then performed an iterative evaluation involving different combinations of the base, engineered and activity parameters. We found that the best-performing model (T_rf3_) was a random forest regressor which utilized T_eng16_ (polynomial regression with T_ac1_, T_ac2_, T_ea_, HR and T_ac1_ + T_grad_) and trial phase (pre-trial baseline, heating, post-trial recovery) as model parameters.

**Figure 5:**
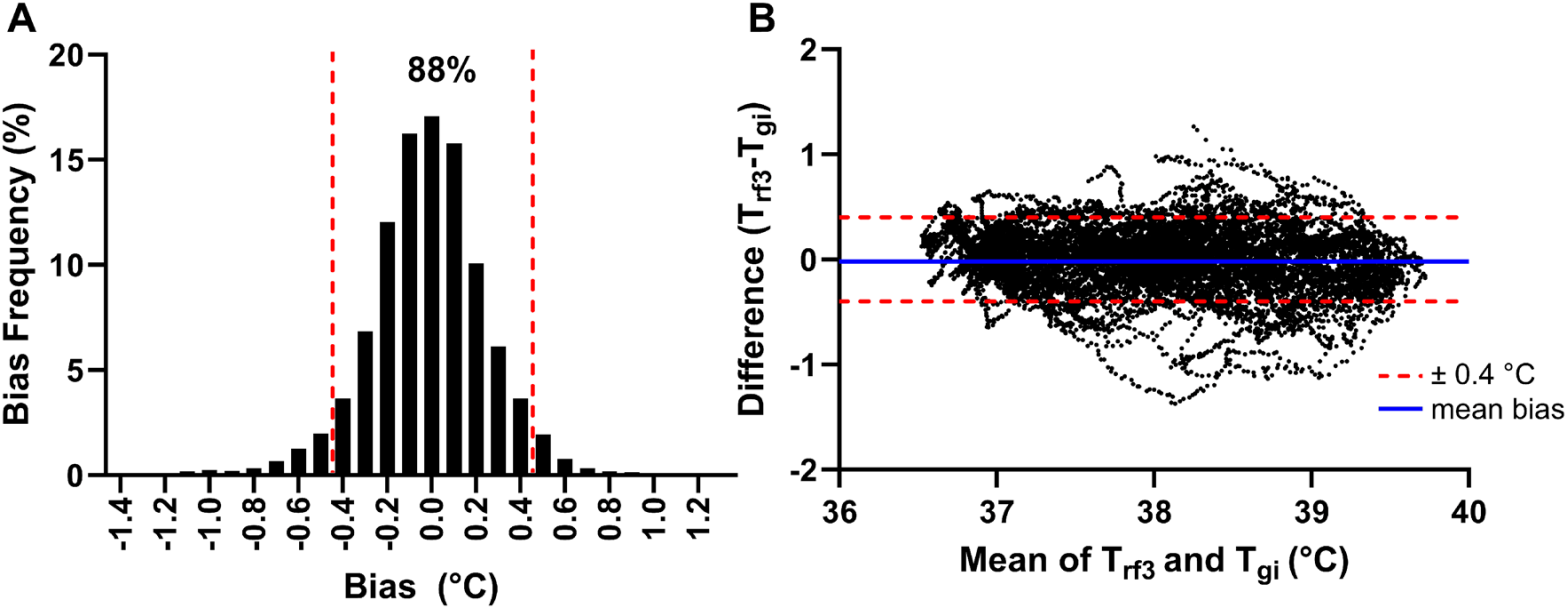
(**A**) Histogram depicting percentage distribution of errors and (**B**) Bland–Altman plots comparing best performing ear-based T_c_ algorithm (T_rf3_) data against the telemetric capsule (T_gi_) across all trials. The solid blue line represents the mean bias error while the red dashed lines represent fixed upper and lower limits of agreement of ± 0.40 °C.

The T_rf3_ model displayed a MBE of − 0.02 ± 0.26 °C, 95% CI of ± 0.52 °C, MAE of 0.20 ± 0.18 °C, MAPE of 0.52 ± 0.46%, and RMSE of 0.27 °C. The MBE for T_rf3_ was within acceptable limits (± 0.27 °C), but PTA ± 0.40 °C (88%) marginally exceeded the predetermined validity criterion (Fig. 5).

### Validity of ear-based T_c_ algorithm during different modes of activity

In order to assess model performance during various activity modalities, the dataset was split into five separate trial phases which comprised of a passive heating, running, walking, pre-trial baseline and post-trial recovery.

**Figure 6:**
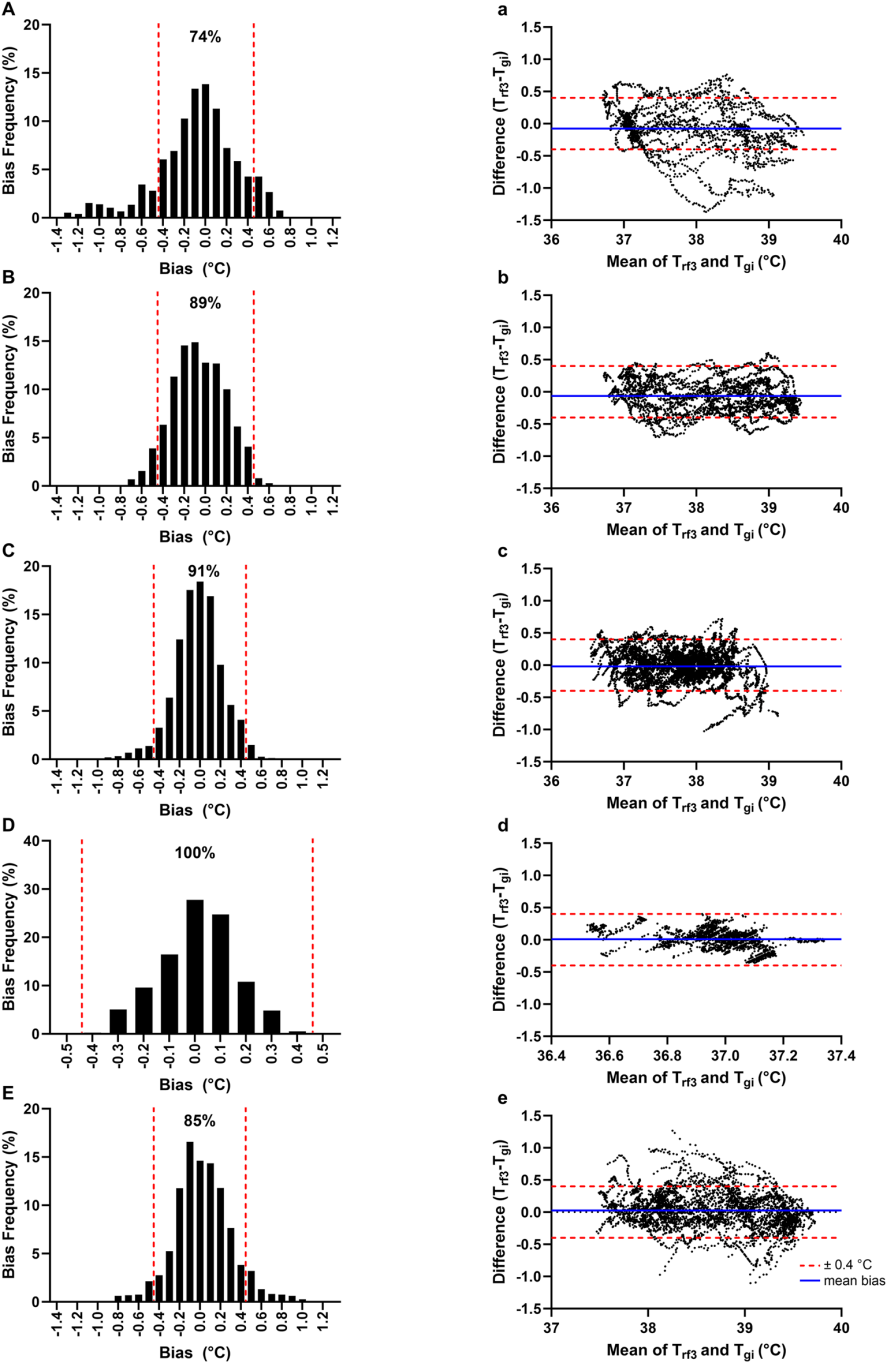
Histogram depicting percentage distribution of errors when comparing best performing ear-based T_c_ algorithm (T_rf3_) data against the telemetric capsule (T_gi_) during the (**A**) passive heating, (**B**) exercise run, (**C**) exercise walk, (**D**) pre-trial baseline and (**E**) post-trial recovery. Bland–Altman plots comparing best performing ear-based T_c_ algorithm (T_rf3_) data against the telemetric capsule (T_gi_) during the (**a**) passive heating, (**b**) exercise run, (**c**) exercise walk, (**d**) pre-trial baseline and (**e**) post-trial recovery. The solid blue line represents the mean bias error during each trial phase while the red dashed lines represent fixed upper and lower limits of agreement of ± 0.40 °C.

The MBE between T_rf3_ and T_gi_ were within the validity criterion during all trial phases: passive heating (− 0.08 ± 0.38 °C), running (− 0.06 ± 0.25 °C), walking (− 0.02 ± 0.23 °C), pre-trial baseline (0.01 ± 0.15 °C), post-trial recovery (0.03 ± 0.28 °C, Fig. 6a–e). PTA ± 0.40 °C was acceptable during pre-trial baseline (100%, Fig. 6D) but exceeded the study validity criterion during the remaining trial phases (all < 95%, Fig. 6A–C,E). The T_rf3_ model exhibited higher accuracy during the running (PTA ± 0.40 °C = 89%, 95% CI  ± 0.49 °C) and walking phases (PTA ± 0.40 °C = 91%, 95% CI  ± 0.46 °C) compared to the passive heating phase (PTA ± 0.40 °C = 74%, 95% CI  ± 0.74 °C).

### Validity of ear-based T_c_ algorithm at different T_gi_ ranges

**Figure 7:**
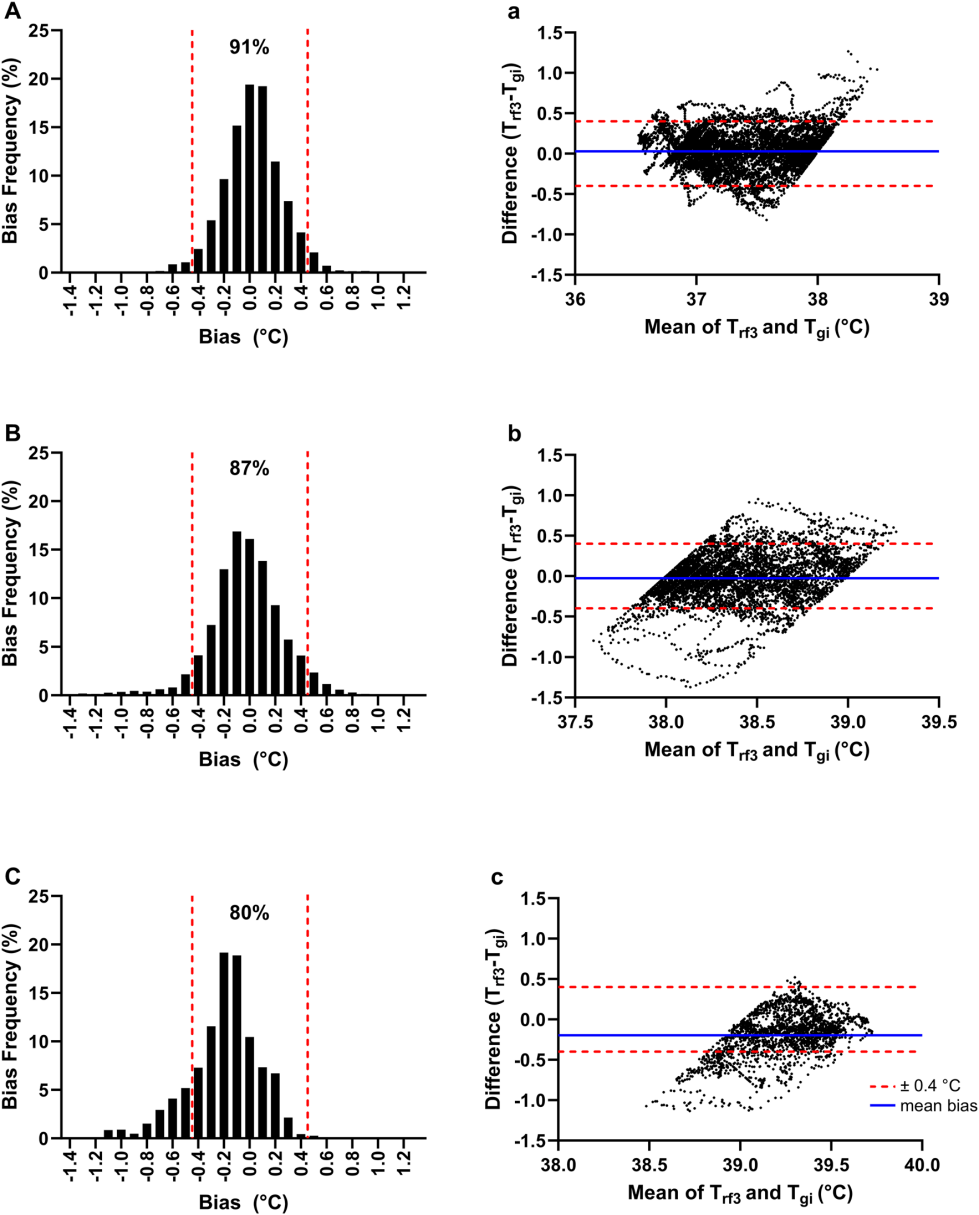
Histogram depicting percentage distribution of errors when comparing best performing ear-based T_c_ algorithm (T_rf3_) data against the telemetric capsule (T_gi_) at (**A**) low (T_gi_ < 38.0 °C), (**B**) moderate (T_gi_ = 38.0–39.0 °C) and (**C**) high (T_gi_ > 39.0 °C) endogenous heat loads. Bland–Altman plots comparing best performing ear-based T_c_ algorithm (T_rf3_) data against the telemetric capsule (T_gi_) at (**a**) low (T_gi_ < 38.0 °C), (**b**) moderate (T_gi_ = 38.0–39.0 °C) and (**c**) high (T_gi_ > 39.0 °C) endogenous heat loads. The solid blue line represents the mean bias error during each trial phase while the red dashed lines represent fixed upper and lower limits of agreement of ± 0.40 °C.

The accuracy of T_rf3_ was assessed at three T_gi_ ranges to determine model validity at different endogenous heat loads: low heat load (T_gi_ < 38.0 °C), moderate heat load (T_gi_ = 38.0 to 39.0 °C), high heat load (T_gi_ > 39.0 °C). The PTA ± 0.40 °C was higher at low (91%) and moderate (87%) heat loads when compared with high heat loads (80%). However, PTA ± 0.40 °C did not meet the study validity criterion at all heat loads (Fig. 7A–C). A similar trend was observed when model sensitivity was assessed across the various T_gi_ ranges (low = 92%, moderate = 79%, high = 70%, Supplementary Table S1). Nevertheless, model accuracy (low = 91%, moderate = 85%, high = 94%) and specificity (low = 90%, moderate = 88%, high = 97%) improved when estimating higher T_gi_ ranges (Supplementary Table S1). The MBE was within the validity criterion at all endogenous heat loads: low (0.03 ± 0.23 °C), moderate (− 0.03 ± 0.28 °C), high (− 0.20 ± 0.27 °C). 95% CI at each heat load are as follows: low (± 0.45 °C), moderate (± 0.56 °C), high (± 0.53 °C, Fig. 7a–c).

## Discussion

We developed an algorithm to predict T_c_ during passive- and exercise-induced heat stress by modifying a commercially available multi-sensor ear-based wearable. In doing so, we investigated whether the inclusion of external auricle temperature (T_ea_) as a model input could enhance the predictive accuracy of the ear-based T_c_ algorithm. T_ac_ markedly underestimated T_gi_ which indicates that it is unsuitable as a sole surrogate measure of T_c_. Inclusion of T_ea_ as a model input improved the predictive abilities of the ear-based algorithm suggesting that T_ea_ can account for environmental influences on the aural canal. The T_rf3_ model (best performing T_c_ model) had individual prediction errors (PTA ± 0.40 °C = 88%, 95% CI =  ± 0.52 °C) that marginally exceeded the study validity criterion (95% PTA within ± 0.40 °C). However, T_rf3_ exhibited acceptable group prediction errors (MBE <  ± 0.27 °C) across all modes of heating. As such, this highlights its potential utility for group-based T_c_ monitoring, with additional refinement needed to extend its applicability to personalized heat strain monitoring.

We observed that T_ac_ significantly underestimated T_gi_ in the present study. Aural canal temperature displayed large negative individual prediction errors (Fig. 3) which culminated in an overall negative MBE (− 1.25 ± 0.86 °C) when compared against T_gi_. This is in line with previous investigations which have reported that T_ac_ measurements were consistently lower compared to gold standard T_c_ references during continuous exercise^16,38^ and simulated work-rest cycle protocols^20,39^. Moreover, T_ac_ measurements derived from our ear-based wearable demonstrated large individual and group prediction errors that markedly exceeded the study’s predetermined validity criteria (Fig. 3). As such, this indicates that T_ac_ should not be employed as a sole surrogate of T_gi_ when used for heat strain monitoring.

Variations in ambient conditions can alter the level of agreement between T_ac_ and T_c_^20^. Hence, several studies have sought to mitigate external environmental influences by insulating the aural canal with a padded ear patch^16^ or medical film^40^. While these strategies are shown to slightly improve the agreement between T_ac_ and gold standard T_c_ references, these approaches are impractical in real-world scenarios. A novel finding in the present study was that T_ea_ can be utilized to account for environmental influences on the aural canal. Inclusion of T_ea_ data as a model input led to a notable improvement in PTA ± 0.40 °C (T_rf1_ = 71%, T_rf2_ = 65%) and narrower 95% CI (T_rf1_ =  ± 0.82 °C, T_rf2_ =  ± 0.94 °C) in T_rf1_ (T_ea_ included in model) relative to T_rf2_ (T_ea_ excluded from model). This suggests that T_ea_ can augment the predictive abilities of an ear-based T_c_ algorithm. Our findings agree with prior work which underscores the importance of including an external temperature sensor to account for environmental effects on the aural canal^21^. It is worth noting that while our approach shares similarities with Nakada, et al.^21^, the external sensor employed in their study directly measures alterations in ambient temperature. In contrast, our T_ea_ sensor derives temperature readings from the skin at the external auricle, offering insights into the heat exchange dynamics between the environment and the auricular region. In doing so, this could provide a valuable physiological perspective into how the external environment influences T_ac_.

Consideration of individual prediction errors is necessary when determining the validity of a T_c_ algorithm for personalized heat strain monitoring^41^. Yet, few published T_c_ algorithms have met the validity thresholds set in the present study^42^. To date, only Nazarian, et al.^43^ have published a T_c_ prediction algorithm that confers an ideal 95% PTA of errors within ± 0.27 °C. Nevertheless, it is noteworthy that their algorithm was developed within a narrower T_c_ range (maximum T_gi_ < 39.0 °C), with treadmill walking employed as the sole activity modality^43^.

We utilized feature engineering and selection to improve the accuracy of the random forest regressor models in the present study. Mathematically transforming and/or combining multiple physiological parameters can generate supplementary model inputs that exhibit enhanced correlations with the intended parameter of interest^44^. Accordingly, our T_rf3_ model displayed an 88% PTA of errors within predetermined thresholds of ± 0.40 °C which considerably out-performed earlier model iterations (T_rf1_ = 71%, T_rf2_ = 65%). Moreover, T_rf3_ conferred a better agreement with T_gi_ when compared with ear-based wearables evaluated in previous research^16,20,45^. Roossien, et al.^45^ validated a commercially available ear-based wearable (Cosinuss° type C-med) and reported underestimations of T_gi_ during rest (− 0.4 ± 0.7 °C), activity (− 1.4 ± 1.5 °C) and recovery (− 1.5 ± 1.2 °C) which were larger than in the present study (Fig. 5). However, Cosinuss° was tested in the field during firefighting task simulations which might have contributed to the poorer agreement observed when compared with our fixed intensity laboratory protocol. When considering a narrower PTA of errors within ± 0.30 °C, T_rf3_ (79%) also surpassed other commercially available wearables such as Kenzen (70%) and the CORE heat flux sensor (40–59%)^33,46,47^. Although the T_rf3_ and Kenzen T_c_ algorithms appear to exhibit a higher accuracy, it is worth noting that the models developed here and in Moyen, et al.^33^ implemented the same dataset for training and validity testing. Thus, further research is necessary to ascertain whether the accuracy of the T_rf3_ model can be maintained when validated across new and independent datasets.

Additionally, comparison between the various heating modalities revealed that T_rf3_ displayed fewer incidences and smaller magnitudes of individual prediction errors when estimating T_c_ during exertional settings relative to passive heating (Fig. 6A–C). Our findings diverged from those presented by Kato, et al.^40^ who reported little difference in 95% CI whilst testing their ear-based wearable during passive heating (± 0.5 °C) and exercise (± 0.4 °C). This discrepancy is likely attributed to methodological differences between the passive heating protocols used in both studies. Notably, Kato, et al.^40^ opted for a lower leg immersion protocol which resulted in comparatively lower levels of heat strain (T_rec_ < 38.0 °C) relative to our study. Furthermore, participants were required to soak up to chest level in the present study, thereby resulting in a closer proximity between the ear-based wearable and the hot water surface. We postulate that radiative heat from the hot water along with cooler external ambient conditions may exert contrasting influences on aural canal and external auricle temperature. In turn, these conflicting signals could potentially lead to a reduction in algorithm accuracy during passive heating. Further work is thus required to better account for these dynamic influences and enhance the applicability of our ear-based T_c_ algorithm during passive heat stress.

Although individual prediction errors were not sufficiently precise for personalized heat strain monitoring, T_rf3_ displayed an acceptable accuracy for estimation of group-based T_c_ responses. Accurate measurement of group-based T_c_ responses could offer valuable information to improve training standards and aid in the estimation of training stimulus when implementing HA protocols^48,49^. HA protocols typically aim to maintain T_c_ above an endogenous thermal criterion of 38.5 °C to elicit an optimal adaptation stimulus^48^. It is thus worth noting that T_rf3_ exhibited an acceptable MBE (− 0.03 ± 0.28 °C) at T_gi_ ranging from 38.0 to 39.0 °C (Fig. 7a–c). As such, this highlights the potential utility of our ear-based T_c_ algorithm to function as a non-invasive tool to quantify group-based T_c_ responses during HA.

## Limitations

The present study was designed to develop and validate our ear-based T_c_ algorithm over a variety of activity modalities and a wide T_gi_ range. As such, we employed continuous passive and exercise heating protocols under controlled laboratory environments to impose adequate environmental and/or metabolic heat stress for elevated T_gi_ readings to be attained. In doing so, we are unable to ascertain whether the T_rf3_ model would confer a similar accuracy when employed in the field. Given that environmental conditions were also tightly controlled in our protocol, the present study design may not have been able to elucidate the true benefits of including a T_ea_ sensor. It is thus crucial for future investigations to train and test T_rf3_ under a wider range of ambient temperatures, fluctuating environmental conditions and during dynamic real-world activities to fully utilize T_ea_ inputs and develop a robust ear-based wearable algorithm^42^. Additionally, aerobically fit participants were recruited due to their enhanced ability to tolerate high endogenous heat loads^50^. Yet, recruitment of a broader participant demographics is necessary in future investigations to assess the applicability of T_rf3_ in other vulnerable populations (e.g. sedentary adults, elderly). Our ear-based wearable T_c_ algorithm was also developed and tested in a male cohort. Given that sex-based differences in wearable accuracy have been demonstrated in previous research^51^, future work to train and validate T_rf3_ in a female cohort is necessary.

## Conclusion

A novel finding in this study was that the predictive abilities of an ear-based algorithm can be enhanced by inclusion of T_ea_ as a model input to account for environmental influences on the aural canal. Despite its promise, T_rf3_ displayed individual prediction errors that marginally exceeded the study validity criterion. However, the T_rf3_ model demonstrated an acceptable accuracy for estimation of group-based T_c_ responses when predicting T_gi_ readings ranging from 38.0 °C to 39.0 °C across all modes of heating. Taken together, T_rf3_ demonstrates potential utility for group-based T_c_ monitoring, with additional refinement needed to extend its applicability to personalized heat strain monitoring. Given the prevalent use of ear-based devices in heat-exposed occupations (e.g. radio communication sets), sports and day-to-day living (e.g. Bluetooth-enabled earbuds), this research seeks to lay the foundation for future development of a wearable ear-based physiological monitoring system that may offer protection in numerous heat-exposed activities (e.g. sports, physical labour) and/or vulnerable populations (e.g. older adults, young children).

### Supplementary Information

Supplementary Table S1.

## Data Availability

All data generated and analyzed in the present study are available from the corresponding author upon reasonable request.
