# Supplementary material for: External auricle temperature enhances ear-based wearable accuracy during physiological strain monitoring in the heat
Source: Sci Rep. 2024 May 30;14:12418. doi: 10.1038/s41598-024-63241-2 (PMC11139936; doi:10.1038/s41598-024-63241-2)
Supplement: Supplementary file 1 — Supplementary Table S1. [file 41598_2024_63241_MOESM1_ESM.docx]

**Supplementary Table S1.** Accuracy, sensitivity, and specificity of the best performing ear-based T_c_ algorithm (T_rf3_) data against the telemetric capsule (T_gi_) at A) low (T_gi_ < 38.0 ℃), B) moderate (T_gi_ = 38.0 - 39.0 ℃) and C) high (T_gi_ > 39.0 ℃) endogenous heat loads.

|  | **T_gi_ range (℃)** | | |
| --- | --- | --- | --- |
|  | < 38.0 | 38.0 – 39.0 | > 39.0 |
| **True positive (n)** | 8902 | 5163 | 1662 |
| **True negative (n)** | 8015 | 10564 | 15738 |
| **False positive (n)** | 927 | 1475 | 463 |
| **False negative (n)** | 748 | 1388 | 729 |
| **Accuracy (%)** | **91** | **85** | **94** |
| **Sensitivity (%)** | **92** | **79** | **70** |
| **Specificity (%)** | **90** | **88** | **97** |
